# A trichostatin A expression signature identified by TempO-Seq targeted whole transcriptome profiling

**DOI:** 10.1371/journal.pone.0178302

**Published:** 2017-05-25

**Authors:** Joanne M. Yeakley, Peter J. Shepard, Diana E. Goyena, Harper C. VanSteenhouse, Joel D. McComb, Bruce E. Seligmann

**Affiliations:** BioSpyder Technologies, Incorporated, Carlsbad, California, United States of America; Cincinnati Children's Hospital Medical Center, UNITED STATES

## Abstract

The use of gene expression signatures to classify compounds, identify efficacy or toxicity, and differentiate close analogs relies on the sensitivity of the method to identify modulated genes. We used a novel ligation-based targeted whole transcriptome expression profiling assay, TempO-Seq®, to determine whether previously unreported compound-responsive genes could be identified and incorporated into a broad but specific compound signature. TempO-Seq exhibits 99.6% specificity, single cell sensitivity, and excellent correlation with fold differences measured by RNA-Seq (R^2^ = 0.9) for 20,629 targets. Unlike many expression assays, TempO-Seq does not require RNA purification, cDNA synthesis, or capture of targeted RNA, and lacks a 3′ end bias. To investigate the sensitivity of the TempO-Seq assay to identify significantly modulated compound-responsive genes, we derived whole transcriptome profiles from MCF-7 cells treated with the histone deacetylase inhibitor Trichostatin A (TSA) and identified more than 9,000 differentially expressed genes. The TSA profile for MCF-7 cells overlapped those for HL-60 and PC-3 cells in the Connectivity Map (cMAP) database, suggesting a common TSA-specific expression profile independent of baseline gene expression. A 43-gene cell-independent TSA signature was extracted from cMAP and confirmed in TempO-Seq MCF-7 data. Additional genes that were not previously reported to be TSA responsive in the cMAP database were also identified. TSA treatment of 5 cell types revealed 1,136 differentially expressed genes in common, including 785 genes not previously reported to be TSA responsive. We conclude that TSA induces a specific expression signature that is consistent across widely different cell types, that this signature contains genes not previously associated with TSA responses, and that TempO-Seq provides the sensitive differential expression detection needed to define such compound-specific, cell-independent, changes in expression.

## Introduction

Trichostatin A is a broad-spectrum histone deacetylase inhibitor that has been characterized extensively *in vitro* [1,2]. Because it modulates the lysine acetylation state of histones and other proteins, TSA is commonly used to alter gene expression [3–6]. TSA-regulated genes are reported in publicly accessible databases such as cMAP [7] (https://www.broadinstitute.org/connectivity-map-cMAP) and GSEA [8] (http://software.broadinstitute.org/gsea/msigdb/index.jsp), making this a useful reference compound for validation of new methods as well as for understanding how gene expression assays can be used for compound classification. Gene expression profiles have been used to classify compound responses and to infer molecular mechanisms of action [3–6] as well as classify and group compounds by comparing profiles with those of previously characterized compounds [9]. Hence, the sensitivity with which a given assay platform delivers differential expression results is important for accurate, functionally relevant results. In a database of responses to small molecule compounds such as cMAP, differentially expressed genes vary in significance and rank across replicate studies, even for a single cell line and exposure. We wondered whether this was due to variability in cell responses, or whether the analysis method lacked sufficient sensitivity or repeatability for robustly detecting TSA responses. To address this question and to determine whether additional genes not previously associated with TSA responses could be identified, we used the novel expression profiling assay TempO-Seq to analyze MCF-7 cells treated with TSA, and asked whether this method is robust enough to augment or build a database of compound responses.

RNA-Seq and its variants are well-established methods for profiling gene expression, providing measurements of the whole transcriptome. However, adoption of RNA-Seq for high throughput compound assessments has been limited because RNA-Seq is costly and requires time consuming RNA purification, quantitation, and cDNA synthesis steps that can limit sensitivity and add variability. In addition to its cost, RNA-Seq data alignment to the whole transcriptome takes significant time and computing resources, which can act as a barrier to widespread use in compound screening and in research where large sample numbers are required. Methods that measure a subset of the transcriptome, referred to as “targeted sequencing”, have been developed to lower the sequencing cost/sample, including target-specific selection from whole transcriptome sequencing libraries [10] and multiplexed amplification of cDNA targets for low complexity qPCR or library preparation [11], but these still require RNA purification and cDNA synthesis, are prone to biases characteristic of the selection method, and are often costly and insensitive.

Two targeted sequencing approaches have been previously described that avoid cDNA synthesis, EdgeSeq [12] and RASL-Seq [13–15]. Both assays target RNA sequences by hybridization to DNA oligos, followed by removal of unhybridized oligos and amplification of the remaining product, either without (EdgeSeq) or with (RASL-Seq) oligo ligation. However, neither platform has been scaled to measure the whole transcriptome. Furthermore, the EdgeSeq platform is tied to a proprietary instrument that can only process 96 samples at a time, and may not be applicable to high sample throughput processing using standard automation. Scaling the RASL-Seq assay to high sample throughput is limited in part by the need to capture the targeted RNA by selecting poly-(A)+ RNA onto a surface for buffer exchanges by washing, which is difficult to adapt to automated processing and limits the assay to samples with intact mRNA. RASL-Seq oligo designs are therefore 3′ biased, and have been limited to splice junctions. In addition, the reported high levels of mis-ligation in RASL-Seq [13–15] consume available sequencing capacity, limiting the number of samples that can be pooled per library.

TempO-Seq (Templated Oligo assay with Sequencing readout) utilizes a different approach to targeted sequencing. Namely, the whole transcriptome TempO-Seq assay targets and measures a specific sequence within each gene, yet measures every gene and isotype in the transcriptome, and does so by directly targeting the RNA contained in crude cellular lysates in a homogenous progressive addition assay. TempO-Seq relies on the hybridization of two “detector” oligos (DOs) to adjacent target sequences such that when properly hybridized they can be ligated. Excess unhybridized DOs are removed enzymatically in a process that is readily scaled and automated, then the ligated DO pairs are amplified to add a sample-specific sequence and the adaptors required for sequencing. Because there is no poly-(A)+ selection, there is no positional bias in target location, so DOs are designed to maximize hybridization specificity. The ligation step also provides specificity for single base differences, making even highly homologous genes distinguishable. The unique biochemistry of TempO-Seq also eliminates mis-ligation and assay background, making the whole transcriptome content possible, and allowing precise dose-response and single-cell level measurements while maximizing sequencing flow cell productivity. In addition, the signal from highly abundant transcripts can be attenuated individually in a way that allows accurate back-calculation of the unattenuated read counts while saving sequencing read utilization. Importantly, the sequencing process delivers only already known ligated DO sequences, so there is no complex bioinformatic analysis; the output of the assay is a simple table with expression levels of each gene in each sample.

Using TempO-Seq to profile expression in MCF-7 cells treated with TSA, more than 9,000 differentially expressed genes were detected with a p_adj_ <0.05, consistent with the large change in expression expected from this HDAC inhibitor. When used to query the cMAP database, these genes overlapped the TSA profiles for MCF-7 cells, but also overlapped the TSA profiles for HL-60 cells and PC-3 cells. Derived from breast, liver, and prostate cancers, respectively, there are substantial differences in basal gene expression between these three cell lines, and many genes are TSA-responsive in each. However, the overlapping profiles suggest that TSA may induce a specific set of genes independent of cell type, and may therefore have a more specific effect on gene expression than has been generally recognized. Analysis of the top 5% differentially expressed genes in cMAP identified 43 pan-cell type TSA-responsive genes. Using TempO-Seq, these were confirmed as TSA-responsive in MCF-7 cells. Treatment of MCF-7 cells, PC-3 cells, and HL-60 cells in 3 differentiation states revealed 1,136 differentially expressed genes in common. 785 of these were not reported to be TSA-responsive in the Comparative Toxicogenomics Database [16], suggesting that these are novel members of a cell type-independent TSA signature. These results suggest that TempO-Seq offers the sensitivity and precision needed to discover new compound/gene associations and to support development of new or updated compound-response databases.

## Materials and methods

### Reagents, RNAs, and cells

TempO-Seq reagents are commercially available from BioSpyder Technologies, Inc. These are proprietary, and consist of a 2X Lysis Buffer for making cell lysates or to complement purified RNAs, a DO Pool for measurement of targeted RNAs, Annealing, Nuclease and Ligation reagents, Amplification reagents for use with custom dual indexed adapters for Illumina sequencing instruments, and a custom Index 1 sequencing primer. Other reagents (PBS, ethanol, TE, purified water) were obtained from VWR or Sigma Aldrich. Libraries were purified using the NucleoSpin Gel and PCR Clean-up kit (Macherey-Nagel cat # 740609.50). Sequencing was performed by service providers using Illumina MiSeq, NextSeq 500 or HiSeq 2500 instruments. Sequencing quality was equivalent across these instruments.

Total RNAs for TempO-Seq assay testing included the MAQC Universal Human Reference RNA (Agilent, cat#740000), MAQC Human Brain Reference RNA, and a selection of human tissue total RNAs (Thermo Fisher Scientific, cat # AM6050 and others). Synthetic RNAs for testing absolute sensitivity and fold change were the ERCC ExFold RNA Spike-In Mixes, obtained from Thermo Fisher Scientific (cat # 4456739). For the RNA-Seq cross-platform comparison, MCF-7 and MDA MB 231 cells were lysed according to the TempO-Seq protocol and RNA was purified using TRIzol (Thermo Fisher cat # 15596026). Total RNAs were assayed in both the whole transcriptome TempO-Seq assay (12 replicates each) and in RNA-Seq (6 replicates each), using ribosomal RNA depletion (KAPA Stranded RNA-Seq with RiboErase kit, Kapa cat # KK8483).

Cell lysates were made from MCF-7 or MDA MB 231 cells grown in DMEM with 10% FBS, washed once with 1X PBS before lysis. Cells were lysed at ~2,000 cells/μL in 1X TempO-Seq Lysis Buffer in PBS by incubation for 10 minutes at room temperature, followed by storage at -80°C. Trichostatin A (TSA) was sourced from Sigma Aldrich (cat # T8852) and dissolved in DMSO for the compound treatment titration, ranging from 10 nM to 100 μM TSA in 0.1% DMSO in 3-fold increments. For profiling TSA responses across cell types, MCF-7, PC-3, and HL-60 cells (undifferentiated or differentiated for 5 days with DMSO or with all-trans retinoic acid), were exposed to 1 μM TSA in 0.1% DMSO or DMSO alone for 6 hours before washing with PBS, cell lysis and storage at -80°C.

### Detector oligo design

TempO-Seq DO pairs were designed to target a 50 nt sequence per gene, with each DO targeting 25 bases. In cases where not all transcripts from a given gene could be monitored by a single pair, additional DOs were included. Detector oligo location was 5′ of the most common stop codon, to avoid complications in interpretation due to alternative poly (A) site usage. DOs were designed without reference to splice junctions, and 72% of the DOs in the Human Whole Transcriptome pool were intraexonic. DO designs were scored using a proprietary algorithm based on transcriptome uniqueness, kinetic parameters and simple repeats, and to optimize ligation efficiency and specificity. DOs were qualified by functional testing using denatured gDNA as an equimolar target source for intraexonic DOs, as well as a variety of human reference RNAs and tissue total RNAs.

The Whole Transcriptome DO pools for the studies described here consisted of 20,629 or 21,111 DO pairs targeting 18,886 or 19,300 genes. Assays of lower complexity measuring fewer genes or other content were used for some experiments, including dedicated DO pools measuring the synthetic reference ERCC RNAs, DO pools for cell-specific RNA detection, DO pools for showing context independence, and DO pools for attenuation studies. The ligated DO gene-specific sequences are provided in the processed data files in GEO accession # GSE91395. For highly abundant transcripts in the synthetic reference ERCC RNA pools, attenuators (nonfunctional DOs that compete with functional DOs) were added into the ERCC DO pool to attenuate their signals by a known ratio per RNA transcript.

### Data analysis

After sample demultiplexing using the default Illumina sequencer and bcl2fastq settings, FASTQ files were aligned to the ligated DO gene sequences using Bowtie, allowing for up to 2 mismatches in the 50 nt target sequence. The data analysis pipeline (TempO-SeqR, BioSpyder Technologies, Inc.) delivers graphical and tabular summaries of total/mapped and unmapped reads, a dendrogram of sample clustering, differential expression and PCA tools, and a flat file of read counts per DO pair per sample. For whole transcriptome assays at 4M reads per sample, data alignment took about 30 seconds per sample on 16 CPUs. The data are shown as raw or normalized read counts for comparing replicates. Normalization for this purpose was done by scaling the data to the average of the total reads per sample. For differential expression analysis, data normalization and adjusted p-value [17] scoring was done using the DESeq2 package in R [18].

## Results

### Assay biochemistry

**Fig 1:**
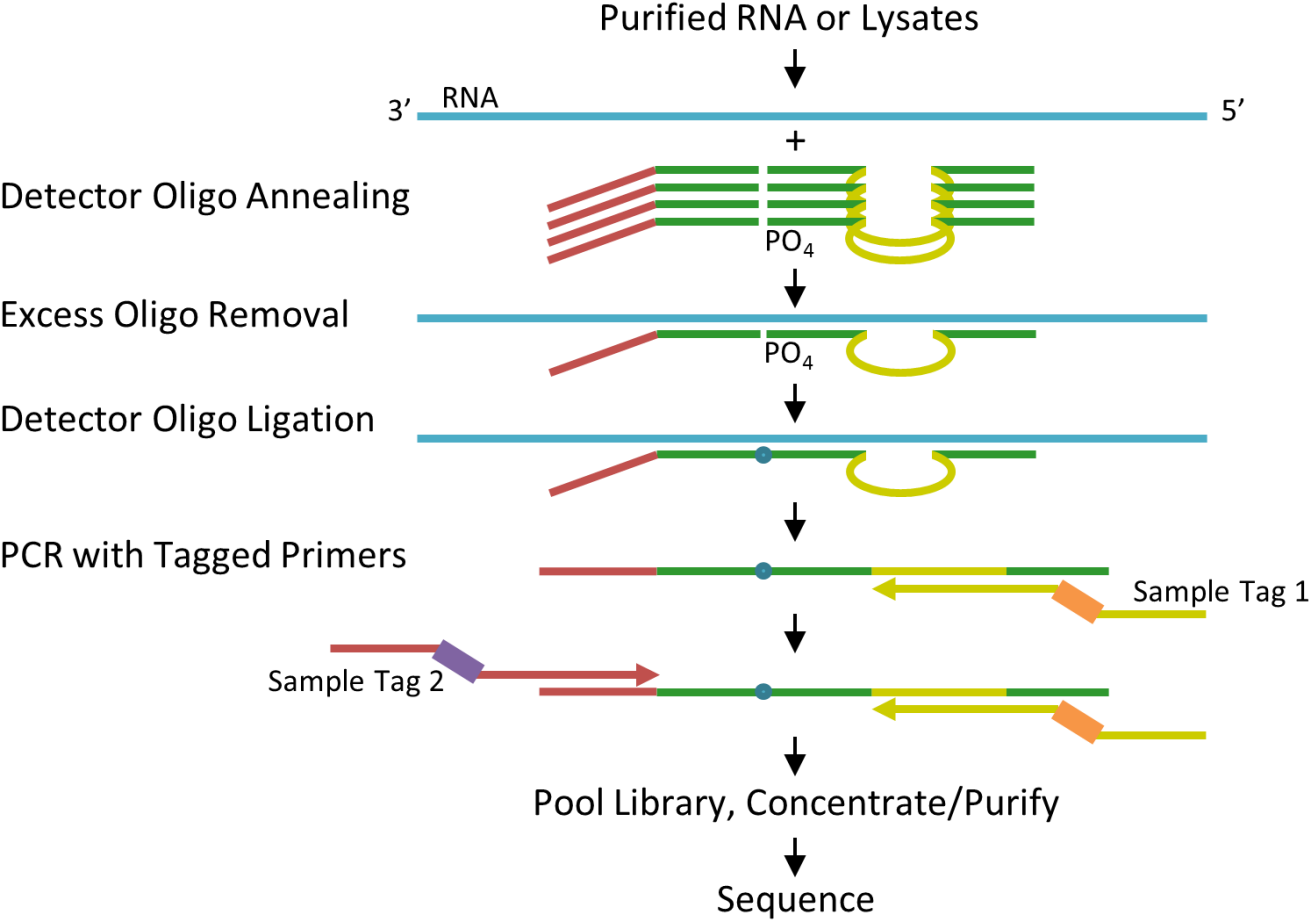
TempO-Seq biochemical scheme. RNAs are targeted by annealing to DOs that contain target-specific sequences (green) as well as primer landing sites (red and yellow) that are shared across all DOs. Excess oligos are removed by a 3′ exonuclease, then the hybridized oligos are ligated and amplified using primers that contain sample tag (index) sequences (orange and purple bars), and adaptors required for sequencing. The amplified assay products are pooled for a library, purified/concentrated and sequenced.

The TempO-Seq biochemical approach and protocol steps are illustrated in Fig 1. The DOs each contain a 25-base sequence that hybridizes to the specific targeted RNA sequence (shown in green). All the DOs share the same primer landing sites (shown in red and yellow), so although the assay can consist of ~20K different DO pairs for 20K different genes, the amplification is single-plex with respect to the PCR primers because a single pair of “universal” primers (a single forward primer and a single reverse primer) amplify every ligated DO pair in the assay. Since the excess oligo removal depends on a 3′ exonuclease, the upstream DO is protected by annealing an additional 25 nt sequence to the target RNA beyond the primer landing site. After annealing the DOs to the target RNA in the sample, subsequent reagents are added, diluting out the prior reagents to ensure enzyme activity (nuclease, ligase, polymerase) is not inhibited. Excess DOs are removed by digestion with a 3′ exonuclease, followed by DO ligation to create an amplifiable template. Amplification is carried out with the universal PCR primers that contain a sample index sequence and the adapter for Illumina instruments. The primers used for each sample have the same universal primer sequences, but a sample-specific index sequence (Fig 1, orange and purple), so that the amplified products contain sequence tags that distinguish the results in one sample from the results in every other sample. The primers used here were validated for pooling up to 384 samples, but this dual 9-mer indexing strategy permits pooling many thousands of samples in a library.

Briefly, the protocol consists of 1) mixing 2 μL of sample in 1X Lysis Buffer with 2 μL of an Annealing Mix that contains a pool of DOs and Annealing Buffer, heating the mixture to 70°C and ramping to 45°C over 50 minutes, 2) adding 24 μL of a Nuclease Mix and incubating at 37°C for 90 minutes, 3) adding 24 μL of a Ligase Mix and incubating at 37°C for 60 minutes followed by 80°C for 15 minutes, and 4) transferring 10 μL of the ligated products into 10 μL of a 2X Amplification mix with sample-specific indexed PCR primer/adapters. Amplification is done in 2 phases, with 6 cycles at an annealing temperature of 54°C and 16 cycles at 66°C. All steps and incubations can be carried out in a PCR thermocycler, or suitable incubator. Assay yield can be monitored in a real-time cycler, as the Amplification mix contains a green fluorescent dsDNA dye. Because the relative abundance of signal among targets is set early in the PCR, the number of cycles has little impact on profiles.

### Repeatability

**Fig 2:**
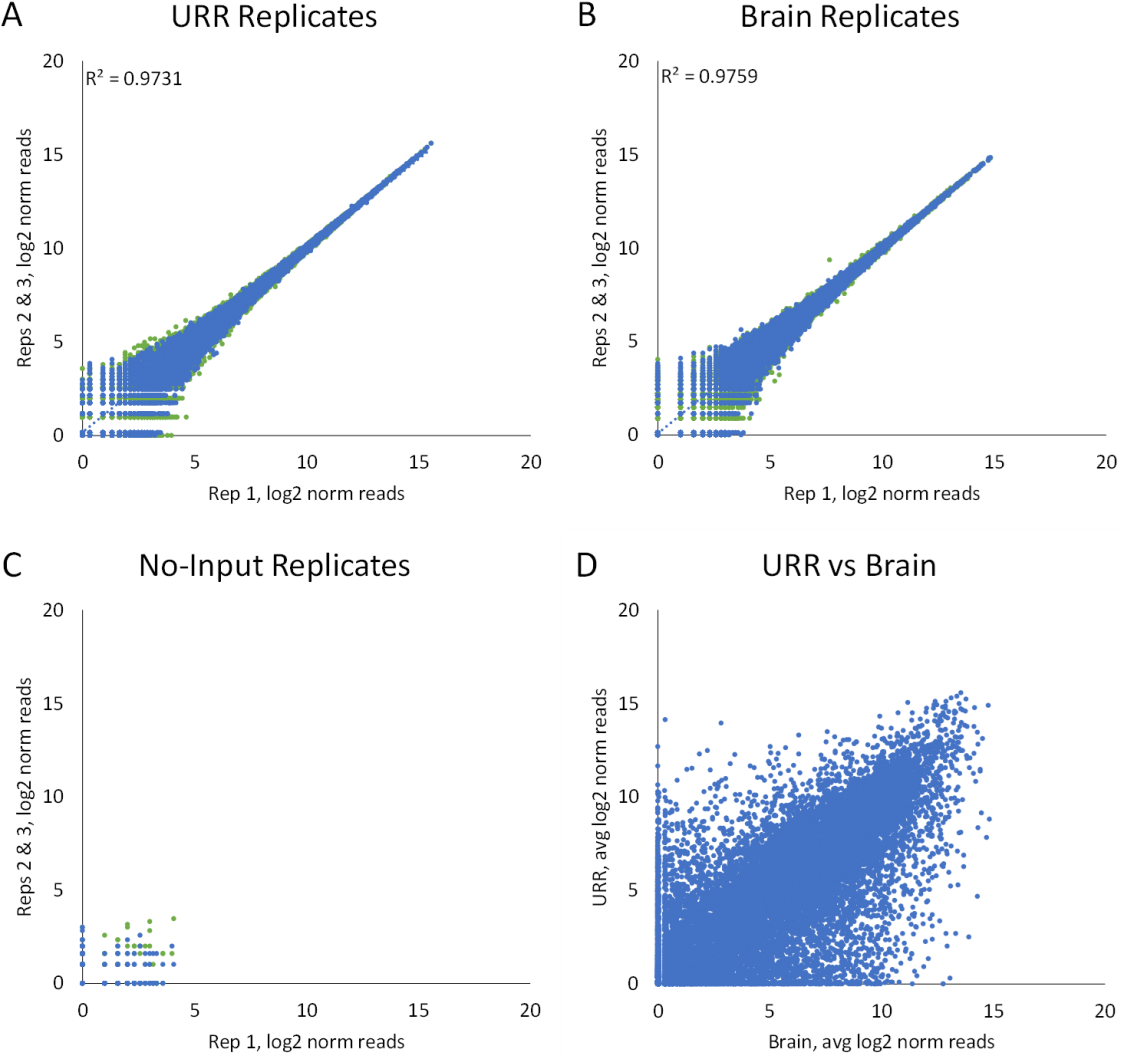
Assay repeatability, background and differential expression. Reference RNAs (100 ng each) were run in the Whole Transcriptome TempO-Seq assay in triplicates of Universal Reference RNA (A), Reference Brain (B), or no RNA input (C). Data were normalized by scaling to the average of the replicates by sample type. Correlations (R^2^) between technical replicates of RNA were 0.97 (URR) and 0.98 (Brain). No-input sample total reads were 0.07% of the reads for Brain and 0.10% of the reads for URR. (D) Differential expression between URR and Brain is shown.

The Whole Transcriptome TempO-Seq assay exhibits excellent repeatability, with R^2^ values for total RNA technical replicates over 0.97 at a read depth of 7.2M per sample. Triplicate assays for 100 ng Universal Reference RNA (URR) and Human Reference Brain RNA (Brain) are shown in Fig 2. Under these conditions, the proportion of reads that map correctly to the expected ligated DO sequences is 85–90%. This correlation between technical replicates in a single sequencing run is very similar to the correlation between a single sample in two different sequencing runs (R^2^ = 0.96, S1 Fig, Seq Run Repeatability), suggesting that the bulk of replicate variability comes from sequencing. The assay is also characterized by very few reads for no-input negative control samples (Fig 2C), with the number of reads for no-input amounting to 0.1% of the reads for the positive samples. Examination of the unmapped reads shows that these consist primarily of partial sequences, rather than mis-ligation. The frequency of mis-ligation is < 0.1%, in contrast to previous ligation-based assays, where mis-ligation can reach 20% or more [13,14]. Fig 2D shows the substantial differences in expression observed between these reference RNAs. Using the DESeq2 package in R, ~45% of all transcripts were differentially expressed between URR and Brain in this experiment at a p_adj_ value <0.05. The p_adj_ is a rigorous method that reduces the false positive rate, and thus differentiates true differentially expressed genes from false positives [17].

### Sensitivity

**Fig 3:**
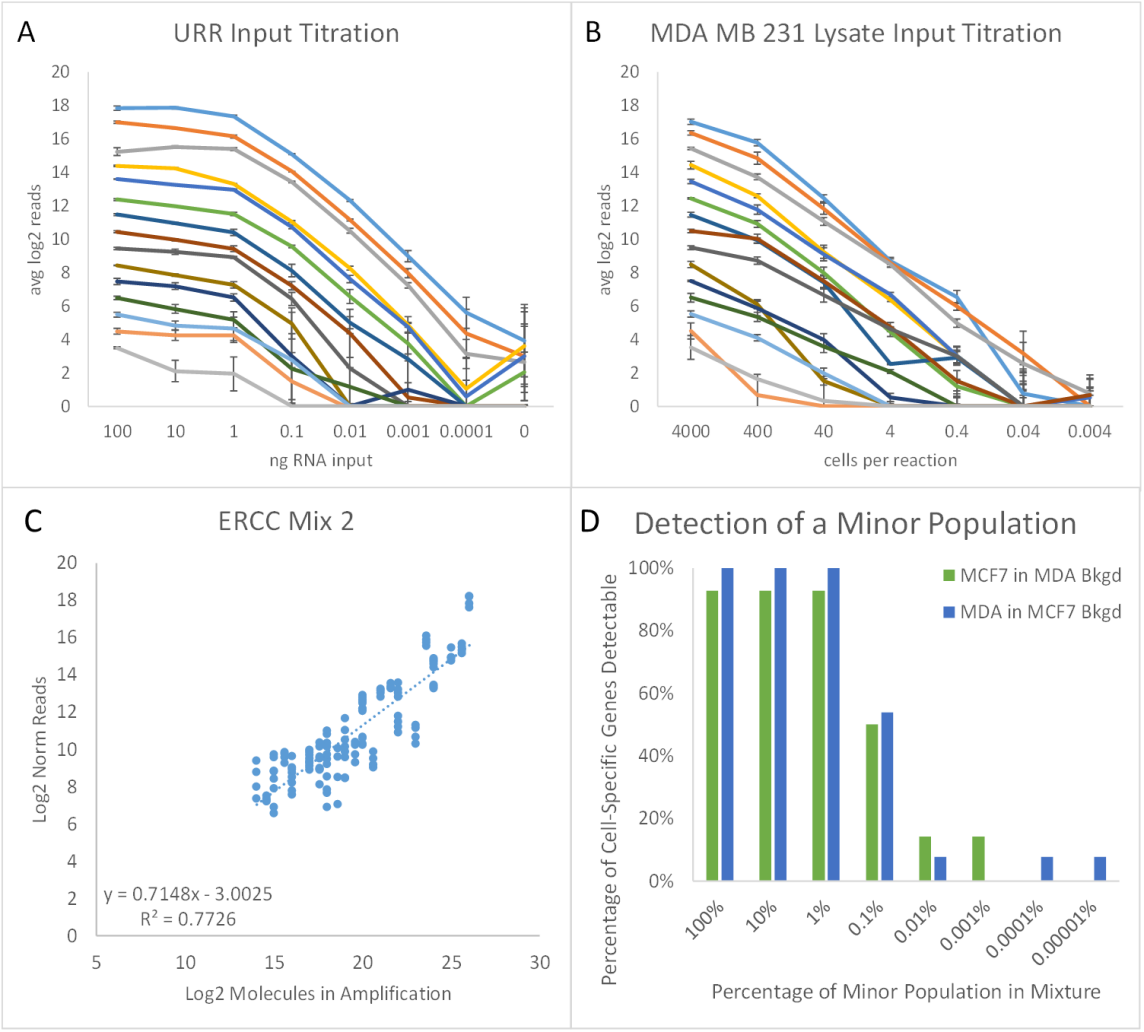
TempO-Seq assay sensitivity. (A) URR RNA was diluted in 10-fold steps with input of 100 ng down to 0.1 pg total RNA, plus no-input, in triplicate. Error bars indicate 1 standard deviation. Each color indicates a different gene, selected across the dynamic range. (B) MDA MB 231 cell lysates were diluted in 10-fold steps, for a range of 4,000 down to 0.004 cells in the assay. Genes were selected as for (A). (C) Mix 2 of the synthetic reference ERCC ExFold RNA Mixtures was diluted in 10-fold steps from 1x10^-3^ down to 1x10^-6^ of the supplied stock in URR as carrier, then assayed using a detector oligo pool specific for the ERCC RNAs. Average reads per sample ranged from 3.6K for the 1x10^-6^ dilution to 340K for the 1x10^-3^ dilution. Results from the 1 x 10^−5^ dilution are shown. (D) MDA MB 231 cells were diluted in 10-fold increments into a constant background of MCF7 cells (blue bars), or MCF-7 cells were diluted into a constant background of MDA MB 231 cells (green bars), then lysed and assayed for cell-specific transcripts. Of the 13 and 14 genes monitored, respectively, the fraction that were significantly above background is shown for each cell dilution. Read depth ranged from 3.6M/sample for 100%, 299K for 0.1%, and down to 64K for 0.00001% for both titrations.

The ability to measure low abundance transcripts is important for establishing and using expression signatures. Assay input sensitivity was assessed by titrating down the total RNA added. Fig 3A & 3B show a representative selection of transcripts across the assay’s dynamic range vs an input titration of URR RNA from 100 ng down to 0.1 pg, two orders of magnitude less than 1 cells’ worth of total RNA (Fig 3A), or an input titration of MDA MB 231 cell lysates from 4,000 cells down to 0.004 cells in the assay (Fig 3B). The data were scaled to the average read number by input level, and genes plotted were selected arbitrarily, representing the average gene for each log2 bin of reads. The lowest expressed transcript thus represents an average abundance of 11 read counts at the highest sample amount. Note that at least half of all genes were still detectable at single cell levels for both purified RNAs and lysates.

The limit of detection is dependent on sequencing depth. In these libraries, the amplification was saturated at the highest inputs, but not at the lower input levels (Fig 3A, note the plateau). The same volume from each amplified sample was pooled to allow comparison of the different input levels, resulting in a range of 2K to 2.1M total reads per sample for purified RNAs and 2K to 13M for lysates (S1 Table). Increased sensitivity for low abundance transcripts at the lower input levels could have been enhanced by increasing the relative amount of amplified product pooled into the library.

To determine the absolute sensitivity of TempO-Seq, we used the ERCC ExFold RNA Spike-In Mixes: synthetic RNA pools prepared in mixtures of known concentrations and ratios for each transcript [19]. These were assayed with a DO pool specific for measuring the ERCC sequences. The ERCC RNA mixtures cover a 6-log range of RNA abundance, but the 7 most abundant RNAs contribute 99% of the mass. This bias in input amounts means that sequencing results would be dominated by these high abundance RNAs, limiting the utility of these mixtures for measuring absolute sensitivity unless the contributions of these 7 RNAs could be reduced. In this experiment, we attenuated the signal from the high abundance transcripts by titrating in non-functional competitor DOs at a fixed ratio per transcript (e.g. a ratio of 4:1 reduces a gene with counts of 50,000 to 10,000). Attenuation is designed to reduce signal from highly abundant targets, but not eliminate them. Instead, the ratio of attenuators to functional DOs is set so that the abundant genes are brought down to about half the level of the highest unattenuated genes. Because the read depth remains high for these genes, there is no impact of attenuation on fold difference measurements (S2 Fig). After data normalization, read counts for the attenuated genes can be corrected by multiplying by the ratio of attenuator to DO for each gene to calculate what their unattenuated read counts would have been. A comparison of results for the same RNA with and without attenuation is shown in S3 Fig, both before and after back-calculation, demonstrating that back-calculated data correlate well with unattenuated data.

To assess the absolute limit of detection, RNA Mixes 1 and 2 were diluted to 1x10^-3^, 1x10^-4^, 1x10^-5^, and 1x10^-6^ of the stock concentrations in a background of 100 ng/test of URR. This dilution series was needed to reduce the target concentrations to approach a lower limit. At the limit of detection, probes began to exhibit increased variability, so Fig 3C shows the relation between reads for probes with less than 60% cv between replicates of the 1x10^-5^ dilution of Mix 2 and the number of molecules present in the amplification step. This level was chosen because the higher inputs (1x10^-3^ and 1x10^-4^) showed assay saturation, and the lower input (1x10^-6^) had high variability due to low reads. The correlation of reads to input was R^2^ = 0.77, consistent with other experiments with these target RNAs. Because of the low assay background, using a single read as the threshold for detection is feasible. For Mix 2, the range of x-intercepts (corresponding to a single read) was 15–19 molecules in the amplification reaction. For both Mix 1 and Mix 2 at this input, the range was from 15 to 72 molecules, with a median of 21 molecules and an average of 32 molecules. Because 19% (10 μL of the 52 μL reaction) of ligated material is transferred to amplification, assay sensitivity is also ~ 20–30 molecules, if one assay is used to supply up to 5 amplifications.

In another approach to determining assay sensitivity, we tested whether a minor population of one cell type could be detected in a background of another cell type. For this experiment, MCF-7 or MDA MB 231 cells were serially diluted into a constant amount of the other cell type, resulting in mixtures with 10-fold decreases in the minor cell population. The mixtures were lysed and assayed in triplicate using DO pools specific for genes that were expressed only in the minor cell population. These cell-specific genes were determined using the whole transcriptome assay, but for this cell dilution experiment, the DO pools contained only the oligos measuring the 14 genes comprising a MCF-7 signature (ARHGAP8, CBS, GREB1, GSTT1, HIST1H1B, HIST1H2BH, HIST1H4E, IGFBP2, IRX2, LAD1, SLC6A14, SULF2, TBX2, and TFF1) or the 13 genes comprising a MDA MB 231 signature (ACSL5, BDNF, C4BPB, CDA, CNN3, EDIL3, ETS1, HMGA2, IFI16, LDHB, MYL9, PTX3, and WBP5). The results show a decrease in the read counts as the minor population was diluted, and are expressed as the fraction of genes in each signature that were over background (Fig 3D). In this case, the background was defined as the average plus 3 standard deviations of read counts in the 0% minor cell population controls. For both cell types, about half the targets could be detected when the minor cell population was 1 cell in 1,000. The abundance of the MCF-7 genes that persist with dilution has been reported to range up to 8,450 transcripts per million reads (TFF1 gene) in the Human Protein Atlas [20]. At a reported 0.5x10^5^ to 1x10^6^ transcripts per mammalian cell [21], this amounts to as few as 8 transcripts detected, consistent with other measures of TempO-Seq sensitivity.

### Specificity

**Fig 4:**
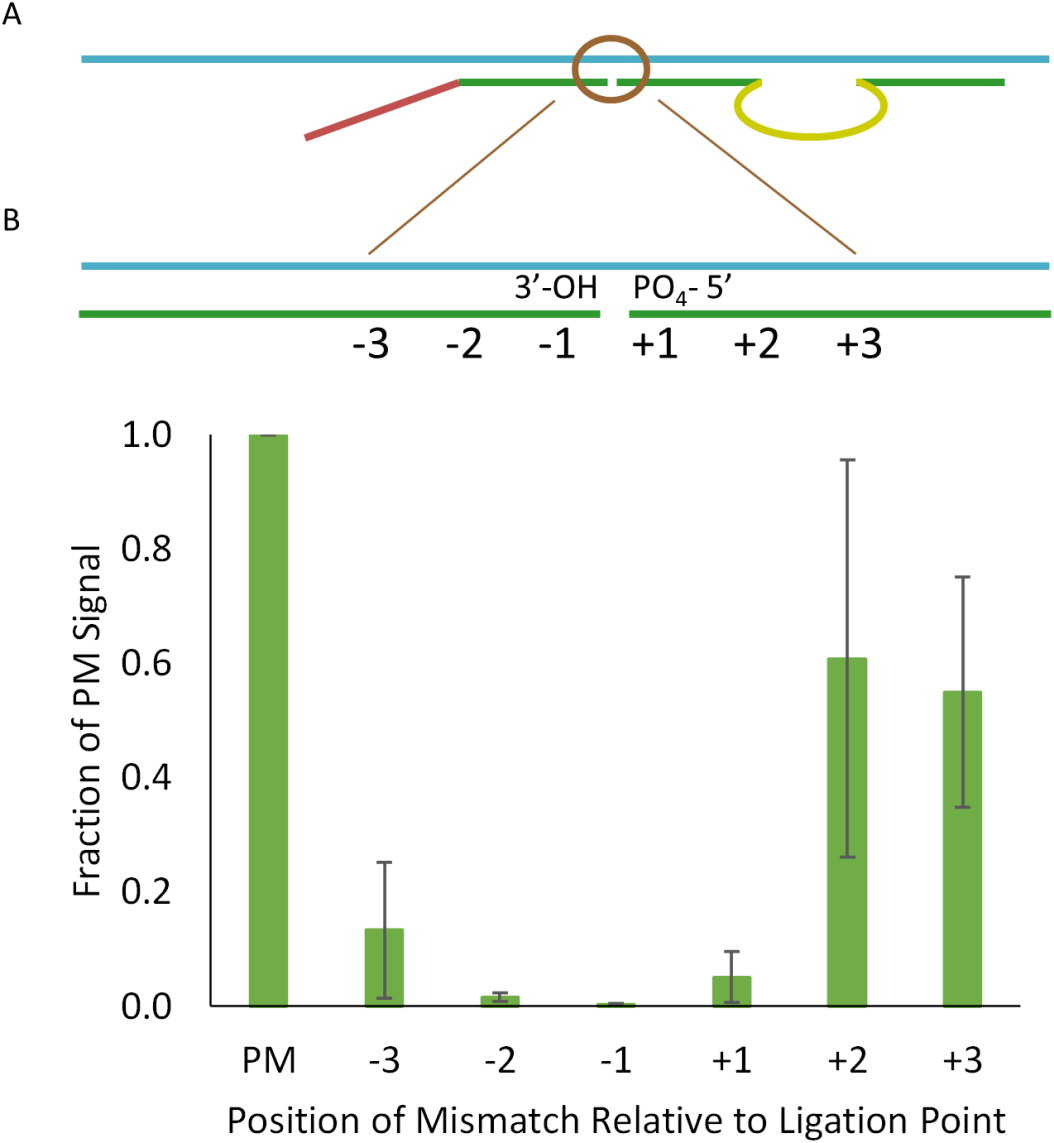
Assay specificity for single base detection. (A) The diagram illustrates the location of single base mis-matches introduced into DOs, numbered on either side of the point of ligation from -3 to -1 at the 3′ end of the downstream DO, and +1 to +3 at the 5′ end of the upstream phosphorylated DO. (B) The average of read counts for 4 targeted transcripts (ACTB, GAPDH, TFF1, TIMP1) was set to 1 for reads from perfect match DOs. The reduction in read counts due to mis-matches is expressed as the fraction of the perfect match reads averaged for 4 genes. Error bars represent 1 standard error. The average read depth was 78K reads/sample.

As a ligation and sequencing-based assay, TempO-Seq should exhibit high specificity. Specificity is critical for accurately measuring the abundance of genes, to avoid false positive signals from cross-reactive targets. This is a particular issue with microarrays where mismatches in hybridization can still result in measurable signals. Although mismatches in either 25-nt target sequence may not detectably affect DO hybridization, a mismatch at the ligation point should be readily detectable. Thus, designing the DOs so that the greatest difference is at the point of ligation was expected to allow TempO-Seq to distinguish between otherwise highly homologous genes or to detect single base differences among RNAs. We tested the specificity of the TempO-Seq assay by introducing single base differences into the DOs within 3 bases on either side of the ligation point and measuring their impact on signal from endogenous high abundance transcripts present in URR. Fig 4A illustrates the position of mismatches in the detector oligos, and Fig 4B shows the effect of these mismatches on read counts. In this experiment, the perfect match/mismatch DOs were added to standard DOs for 20 other targets, later used for normalization. As expected, mismatches closest to the ligation point were most effective at impairing detectable products, with the most disruptive mismatch at the 3′ hydroxyl base averaging 0.4% of the expected read counts, translating into single-base specificity of 99.6%. Thus, the TempO-Seq assay can reliably distinguish between closely related transcripts with single base specificity, consistent with the low level of mis-ligation observed that might otherwise result from ligation on similar RNA target sequences. This level of specificity was critically important for scaling the assay to the whole transcriptome while maintaining independent measurement of each gene without detectable off-target reads. The DO design pipeline incorporates a requirement for absolute sequence specificity between gene targets across the ligation point.

**Fig 5:**
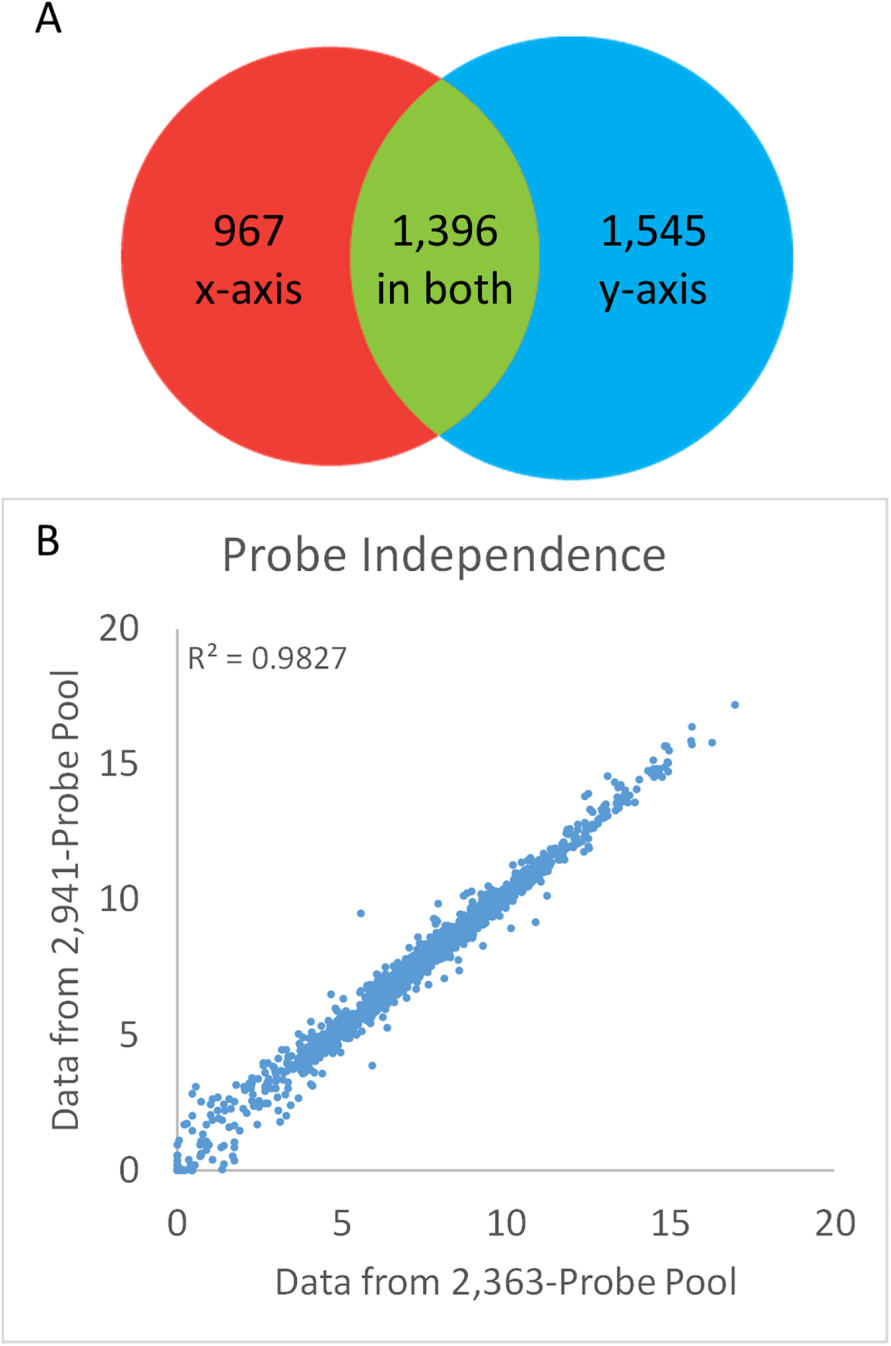
Context independence. (A) The overlap between two DO pools is illustrated. Red indicates the DOs only in the 2,363-plex pool, blue indicates the DOs only in the 2,941-plex pool, and green indicates the 1,396 DOs that are in present in both pools. (B) Read counts for the 1,396 transcripts targeted in both pools are compared (R^2^ = 0.98). Read depth for the 2,363-plex pool was 6.3M/sample, and for the 2,941-plex pool was 4.7M/sample.

The independent measurement of each gene that is a result of the assay’s high specificity is important for content flexibility. If transcript measurements are truly independent in an assay, then the signal for a targeted set of genes should be the same whether they are measured within one or another set of other genes. This was tested with TempO-Seq by comparing data for a common set of probes measured in the context of two otherwise different DO pools (Fig 5). In this case, a DO pool targeting 2,941 transcripts had 1,396 DO pairs in common with another pool targeting 2,363 transcripts, illustrated in the Venn diagram in Fig 5A. Comparing the read counts for the 1,396 targets measured using the two different pools showed a correlation with R^2^ of 0.98 with no significant outliers, demonstrating that the different contexts had little influence, and that expression measurements are independent of context. This feature is particularly valuable for longitudinal studies where DOs may be added or removed, because existing data will still be comparable for the DOs that don’t change. In addition, context independence suggests that initial studies with the whole transcriptome assay will be predictive of DO behavior when they are selected for a smaller gene panel.

### Differential expression

**Fig 6:**
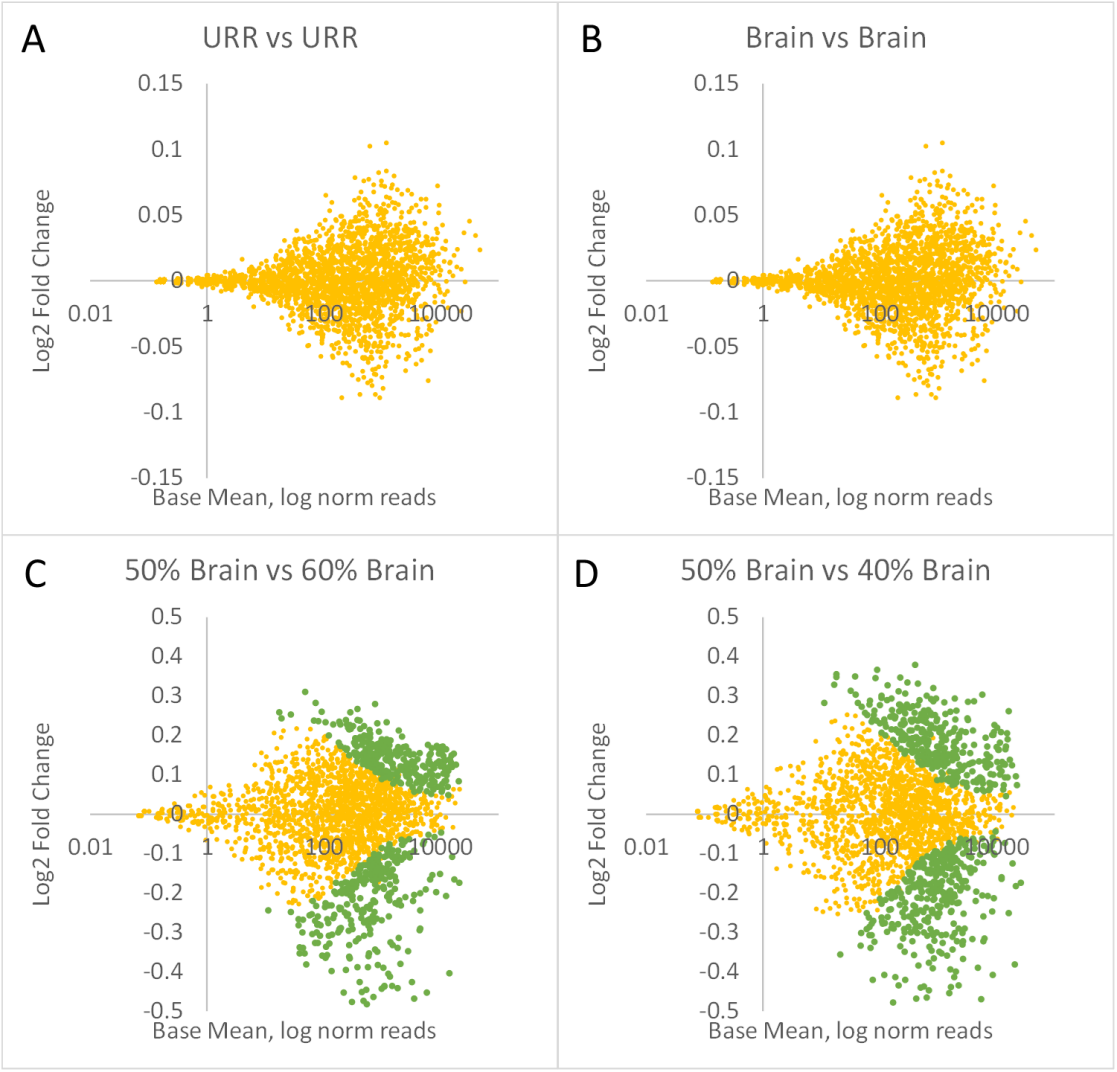
Differential expression sensitivity. Plots show log2 fold change vs log average normalized reads per target derived from DESeq2. Points in green are those significantly different between the two samples (p_adj_<0.05). (A) URR compared to URR, (B) Brain compared to Brain, (C) 50% Brain/50% URR compared to 60% Brain/40% URR, and (D) 40% Brain/60%URR compared to 50% Brain/50% URR. Read depth averaged 1.9M reads/sample.

High assay repeatability, high specificity, and low backgrounds are required for highly sensitive detection of differential expression. To determine how sensitive the TempO-Seq assay is for measuring small changes in expression, we ran technical replicates of the Reference RNAs in an assay of 2,244 genes and compared results both across and within URR and Brain triplicates. Fig 6 shows log2 fold changes vs log mean read counts derived from DESeq2, where green dots indicate genes that are differentially expressed (p_adj_<0.05). Panels A and B show the false positive detection rate, measured by comparing 3 replicates of URR to another 3 replicates of URR (Fig 6A), or comparing 3 replicates of Brain to another 3 replicates of Brain (Fig 6B). The absence of green dots indicates that there were no differentially expressed genes among replicates of the same RNA, as expected. In contrast, Fig 6C and 6D show differential expression between mixtures of RNAs made so that transcript abundance between the mixtures are at most 1.2-fold (20%) different. DESeq2 analysis of 50% Brain/50% URR compared to 60% Brain/40% URR identified 564 differentially expressed transcripts (green dots) at p_adj_<0.05 (Fig 6C). Similarly, DESeq2 analysis of 40% Brain/60% URR vs 50% Brain/50% URR identified 690 differentially expressed transcripts at p_adj_<0.05 (Fig 6D). This result indicates that the TempO-Seq whole transcriptome assay can reliably detect 1.2-fold differences in transcript abundance.

### Verification of differential expression

**Fig 7:**
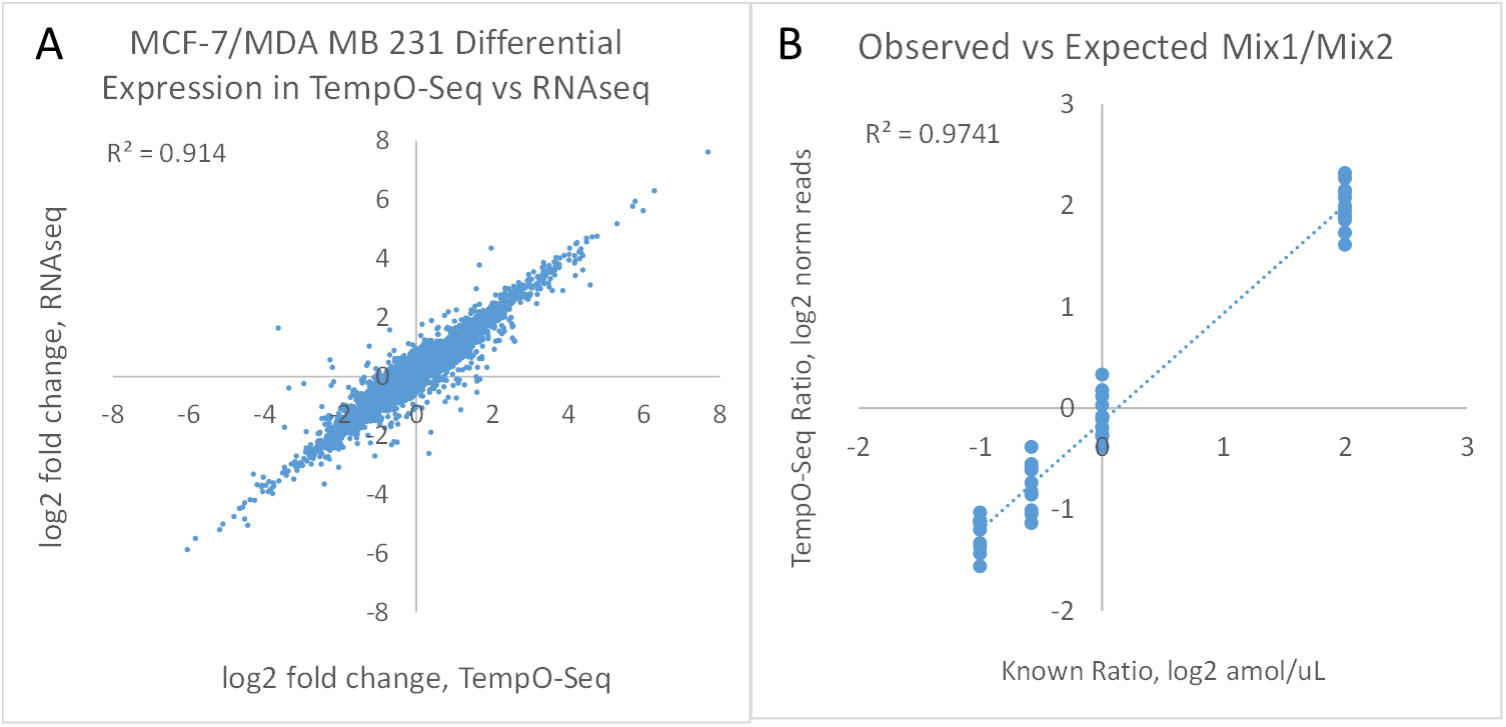
Cross-platform comparison of TempO-Seq and RNA-Seq. (A) Log2 fold changes between MCF-7 and MDA MB 231 RNAs as measured by TempO-Seq (~4 M reads/sample) are compared to those measured by RNA-Seq (~15 M reads/sample) for the 6,500 genes that had over 20 read counts in both cell types on each platform. R^2^ = 0.91. (B) ERCC Mixes 1 and 2 were diluted 1:1,000 and spiked into 100 ng URR, then assayed with a dedicated DO pool. Fold differences in sequencing reads are compared to the fold differences in concentration between the two mixes. Read depth was 340K reads/sample.

To determine whether TempO-Seq accurately measures expected differences in expression, whole transcriptome TempO-Seq data were compared to RNA-Seq data for the same RNA samples prepared from MCF-7 cells and MDA MB 231 cells. This cross-platform comparison was done by determining differential expression in DESeq2 for each assay. A threshold was imposed by removing genes with fewer than 20 reads in either sample on both the TempO-Seq or RNA-Seq platforms to avoid artefactual noise caused by dividing by small numbers of counts. Although a threshold of 20 reads was chosen arbitrarily for platform comparison, the definition of appropriate thresholds for sequencing-based assays is an active area of study [22]. The resulting correlation between log_2_ fold change for the 2 cell lines is shown in Fig 7A, showing an excellent correlation in fold differences in expression measured by TempO-Seq to those measured by RNA-Seq (R^2^ = 0.91). Thus, the differentially expressed genes measured by the TempO-Seq whole transcriptome assay are consistent with those from RNA-Seq whole transcriptome shotgun sequencing, with one quarter of the sequencing space.

The synthetic ERCC RNA Mixes were used to verify the quantitative accuracy of TempO-Seq differential expression (Fig 7B). ERCC Mix 1 and Mix 2 have pre-set ratios between the transcripts, so that log2 fold differences are known. These RNAs were spiked into 100 ng URR at 1:1000 of the stock concentrations and assayed with an ERCC-specific DO pool. The observed fold differences in TempO-Seq were compared to the expected fold differences in the mixes (Fig 7B). The correlation of measured to actual fold differences was excellent (R^2^ = 0.97), confirming that the TempO-Seq assay accurately reports differential expression.

### Treatment response

**Fig 8:**
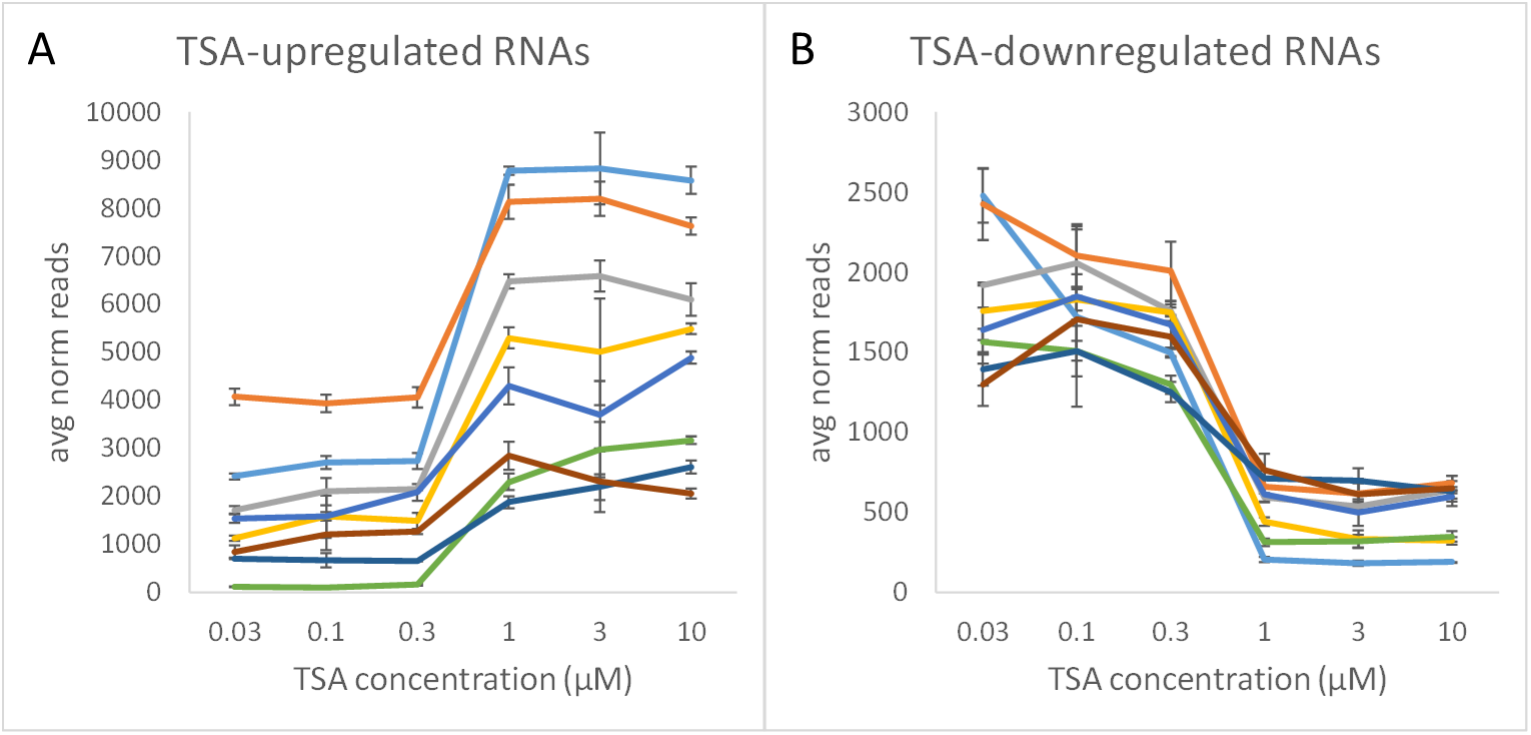
Dose-dependent up- and down-regulated genes in response to Trichostatin A. Triplicate wells of MCF-7 cells were cultured in 384-well microplates to a confluency of ~70% (~5,500 cells/well), exposed to the indicated concentration of TSA in 0.1% DMSO for 6 hours, washed once with PBS, then lysed in 10 μL of 1X Lysis Buffer, and run in the Whole Transcriptome TempO-Seq assay, with an input of 1,100 cells/assay. A representative selection of up-regulated (A) or down-regulated (B) genes are shown, with error bars representing 1 standard deviation of the reads across the triplicates for each gene. Each color line represents an individual gene. The average read depth was 2.7 M reads/sample.

To investigate TempO-Seq sensitivity to detect cellular responses to compound exposure, the whole transcriptome assay was used to monitor dose-dependent gene expression changes in MCF-7 cells treated for 6 hours with Trichostatin A (TSA) at concentrations from 0.03 μM to 10 μM. This is a reference model system for compound response, cell line impact, and treatment duration for which there are considerable data in cMAP that can be used to confirm TempO-Seq results and to test the hypothesis that a significant number of additional TSA-responsive genes otherwise missed in cMAP might be found by a sufficiently sensitive and selective whole transcriptome assay. Fig 8 shows an exemplar set of genes that were either up-regulated (Fig 8A) or down-regulated (Fig 8B) by TSA. Onset of effects occurred at a concentration of 1 μM, consistent with conditions used in cMAP.

**Fig 9:**
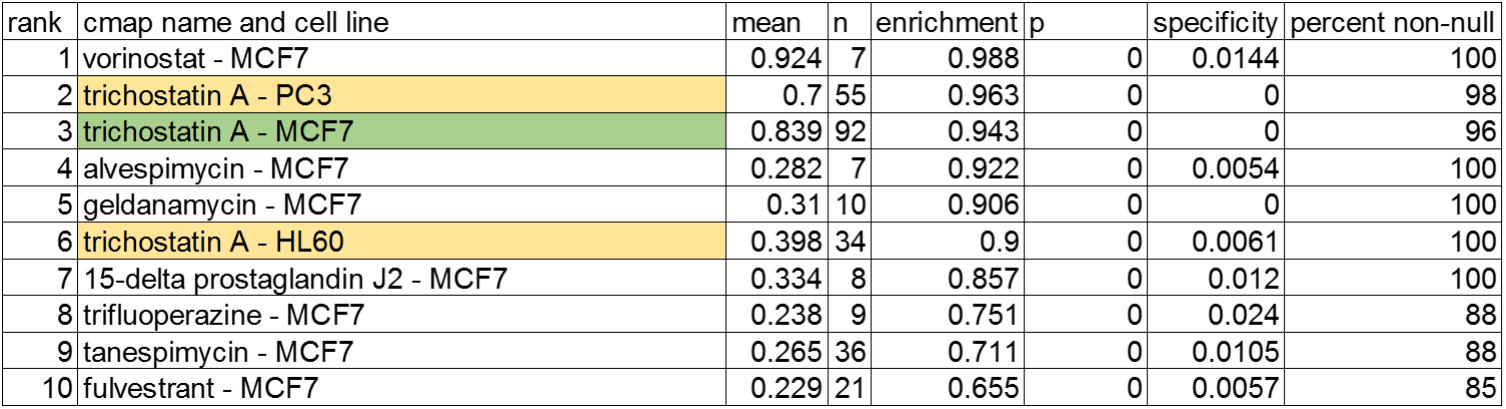
Overlap of Trichostatin A-induced differential expression with profiles in cMAP. The top 10 results are listed from a search of the cMAP database with the most significantly up- and down-regulated genes (150 genes each) reported by TempO-Seq. The matching cell type and treatment is highlighted in green, and TSA treatment of other cell types is highlighted in yellow. Column headings are defined by cMAP and report the output of profile similarity measures (7).

Using DESeq2 analysis, 4,154 differentially expressed transcripts were identified (p_adj_ < 0.05) in response to treatment with 1 μM TSA compared to vehicle control. The most significant 5% of up- and down-regulated genes were used to query the cMAP database of gene expression signatures in response to compound treatments, which includes MCF-7 cell and TSA profiles (Fig 9). Overlapping gene sets included MCF-7 cells treated with Trichostatin A, as expected, and profiles of MCF-7 cells treated with other compounds.

**Fig 10:**
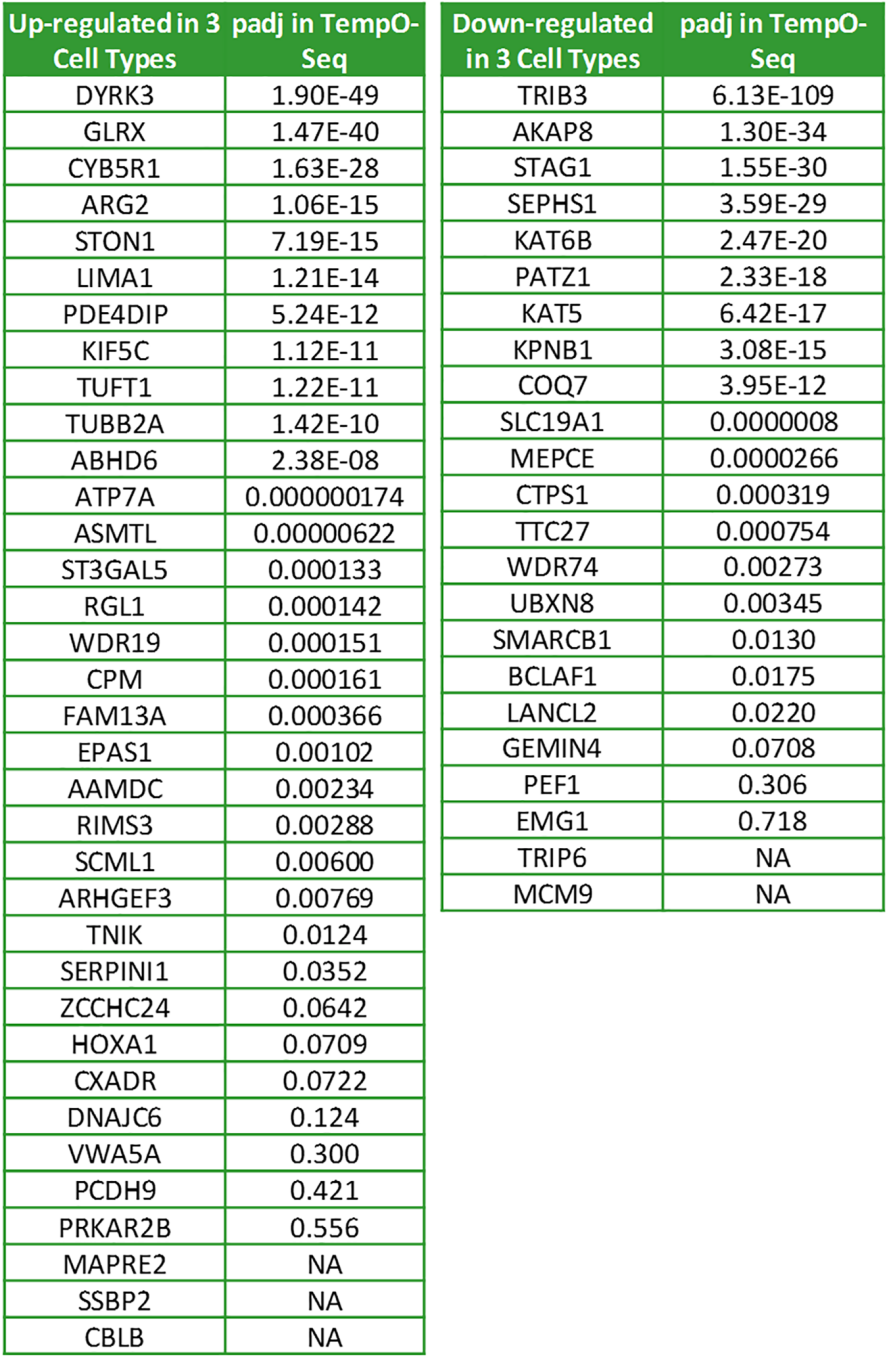
Pan-cell type TSA-responsive expression profile from cMAP data. Genes in the top 5% of those up- and down-regulated due to TSA treatment and in common across the HL-60, MCF-7, and PC-3 cells in the cMAP database are listed, together with the p_adj_ value from the TempO-Seq assay for each gene in MCF-7 cells. “NA” indicates insufficient reads.

The TempO-Seq results also overlapped strongly with the TSA-induced expression profiles in PC-3 cells and HL-60 cells in cMAP, suggesting that there may be a TSA-specific signature across all cells that is independent of baseline expression. To define this signature, gene rankings were downloaded from the cMAP database (http://portals.broadinstitute.org/cMAP/#), and gene IDs and rank were recovered for 69 MCF-7 sample entries, 31 HL-60 entries, and 39 PC-3 entries, all treated with 1 μM TSA for 6 hours, the same treatment conditions used for the TempO-Seq assay. Five of the MCF-7, two of the HL-60, and two of the PC-3 cMAP samples were identified as outliers by PCA and removed, and then a median ranking was computed for each gene ID. The most reliably responsive genes would be at the top and bottom of the rankings, so the top and bottom 5% of ranked genes were examined for those in common across all cMAP data sets. Among the most up-regulated genes, 35 were in common across all cMAP data sets, and among the 5% most down- regulated genes, 23 genes were in common. Of these 58 genes, 43 were scored as significantly differentially expressed in the TempO-Seq data (Fig 10). Of the remaining ten, 5 had p_adj_ values above 0.05, and 5 had <20 reads in the TempO-Seq assay, below the cut-off used. Querying the cMAP database with this set of cell-independent TSA signature genes identified a strong overlap with HDAC inhibitors (S2 Table), as expected. These genes represent the highest and lowest rankings in the cMAP data, but are not a comprehensive list of all TSA-responsive genes in these cells.

**Fig 11:**
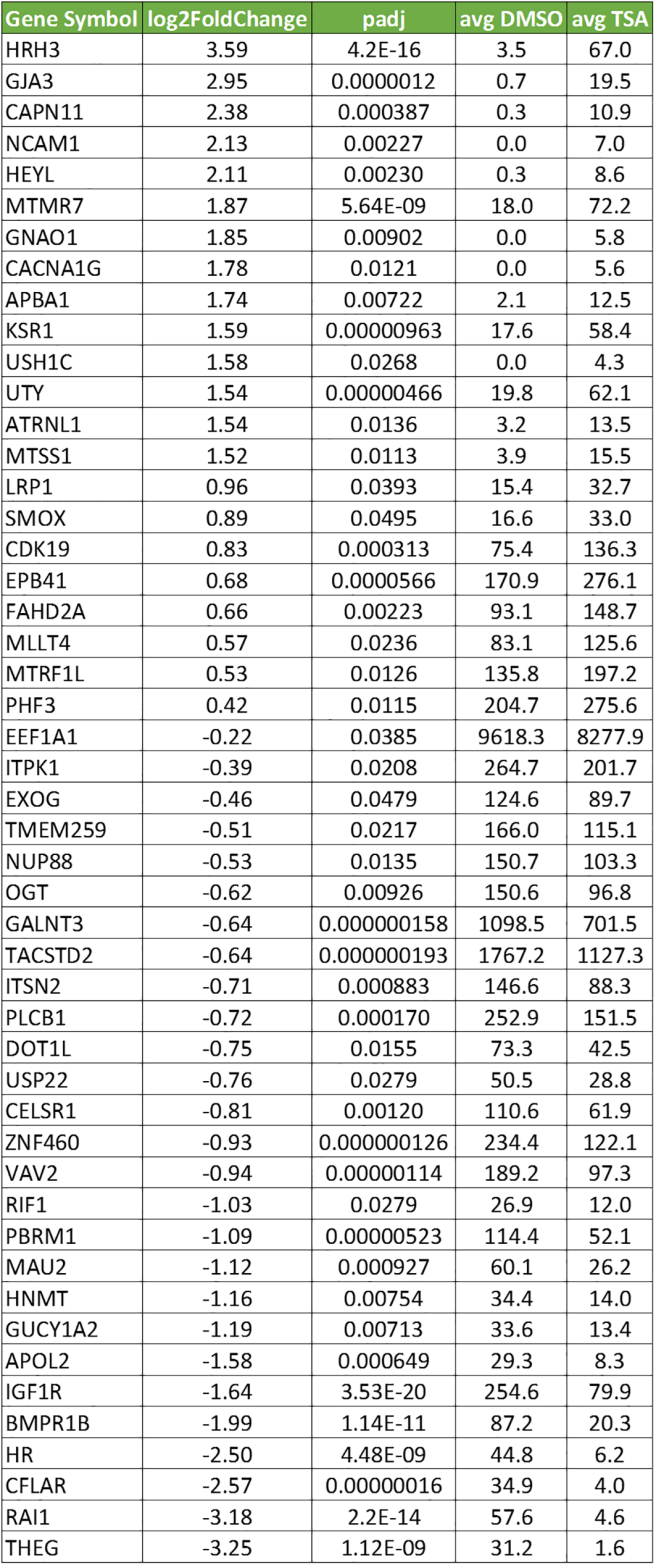
Novel TSA-responsive genes identified with TempO-Seq. Normalized values shown were derived using the DESeq2 package in R.

We observed additional significant TSA-induced responses for genes that were not significant in the cMAP rankings because they fell within 10% of the median ranking for all three cell types, suggesting that TempO-Seq detected additional differentially expressed genes beyond those reported in cMAP. A set of 2,000 genes spanning the middle of the rankings (where differential expression was not significant) had 1,423 genes in common across the 3 cell types. Within these, there were 46 genes that had a p_adj_ < 0.05 in the TempO-Seq results for MCF-7 cells, suggesting these may be co-regulated with the TSA signature but missed by the microarray results that comprise cMAP (Fig 11). Reviewing these genes in the Comparative Toxicogenomics Database [16] revealed that 27 of the 46 genes have not previously been reported to be responsive to TSA. As shown in Fig 11, these genes showed low read counts and/or low fold changes on TSA treatment, suggesting that their detection by TempO-Seq is due to its enhanced sensitivity compared to other expression monitoring technologies.

**Fig 12:**
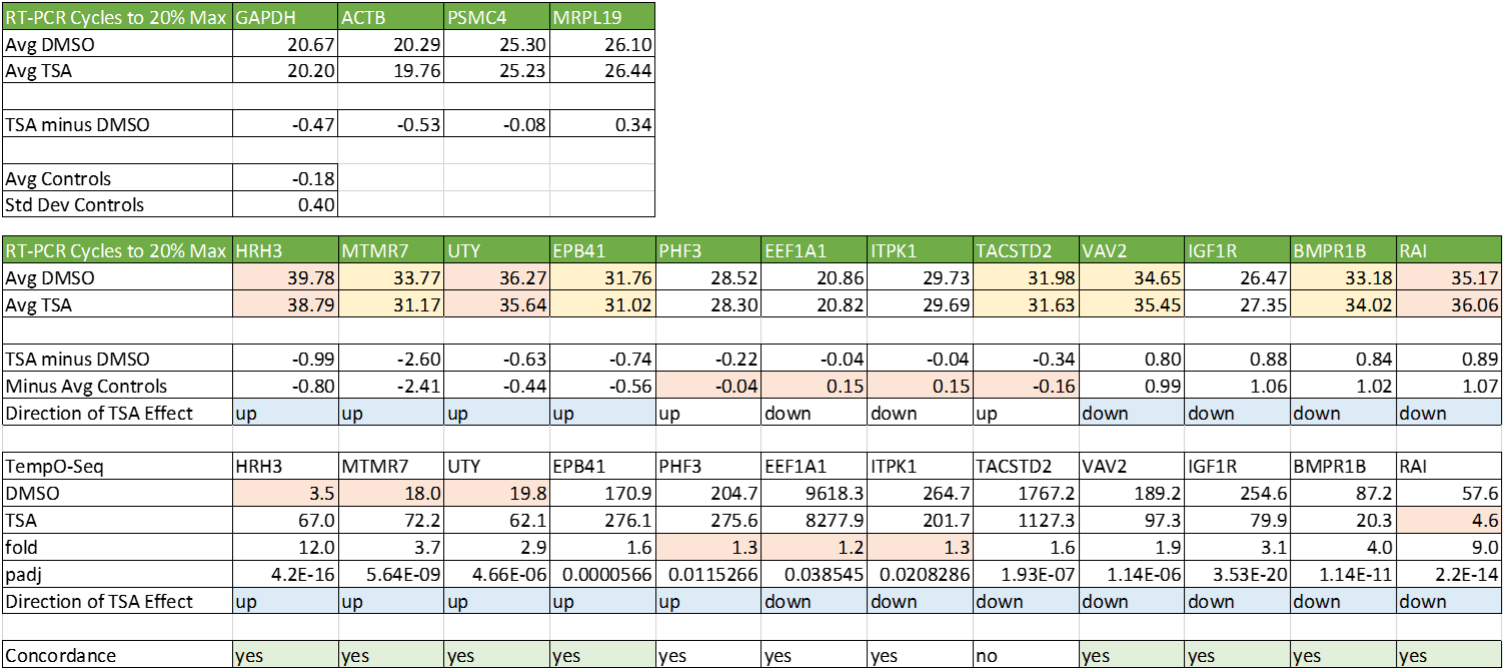
RT-PCR confirmation of selected TSA-responsive genes. For each gene, the average number of cycles needed to achieve 20% of the maximum yield at 45 cycles is listed. Control housekeeping genes are shown above and proposed TSA-responsive genes are shown below. Green highlighting indicates concordance in the direction of the TSA effect. Tan highlighting indicates a limitation impacting sensitivity (high cycle number, small fold difference, low expression, difference in cycle number less than 1 standard deviation of the controls). Yellow highlighting indicates marginal sensitivity (high cycle number). Blue highlighting indicates relative confidence in results to be compared.

Two approaches were taken to confirm these previously unreported TSA-responsive genes: an assessment of the reliability of the TempO-Seq probes for these genes, and RT-PCR analysis. S4 Fig shows the cross-platform comparison from Fig 7, highlighted to identify the results for the novel 46 TSA-responsive genes. The fold differences measured in the cross-platform comparison for these 46 genes correlate well with the rest of the whole transcriptome, demonstrating that the measurement of these transcripts is robust. RT-PCR analysis of these genes was expected to be challenging because of their low abundance and small fold changes. Gene-specific RT primers and a pair of PCR primers were designed for 12 genes that spanned the range and direction of expression changes, as well as 4 housekeeping genes as normalization controls (Fig 12). Of the 12 genes tested, 8 showed changes in expression greater than 1 standard deviation of the control genes. All 8 were concordant with the direction of TSA responses in TempO-Seq. Taken together, these results confirm that the novel TSA-responsive genes detected in TempO-Seq are likely to be valid.

**Fig 13:**
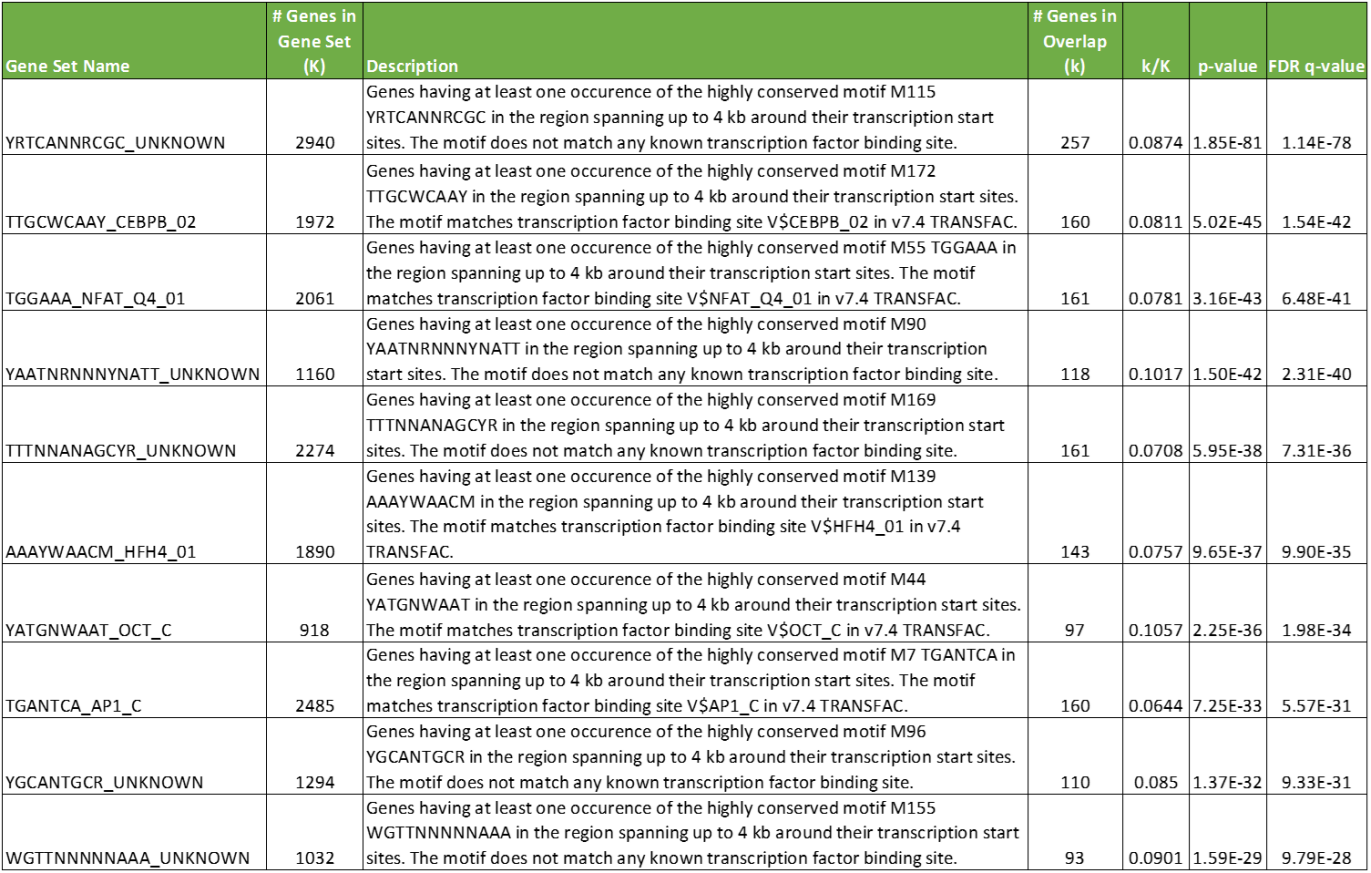
Overlap of pan-cell type TSA-responsive genes with transcription factor motifs. The top 10 gene sets in the transcription factor database searchable in GSEA that overlap with the 1,115 pan-cell type TSA-responsive gene set.

Because the 27 novel TSA-responsive genes were derived from the middle rankings (7,000 to 12,000) of the cMAP data, a comprehensive analysis was likely to reveal additional such genes. TempO-Seq analysis of total RNAs derived from MCF-7, PC-3, and undifferentiated HL-60 cells exposed to TSA under the same conditions as those used in cMAP studies revealed a set of 1,571 genes that were differentially expressed in all 3 cell types, about one third of the total differentially expressed genes in each cell type alone (S3 Table). Expanding this analysis to include HL-60 cells differentiated into neutrophils and granulocytes allowed the derivation of a TSA-responsive profile of 1,136 genes in common among 5 cell types (S4 Table). These were queried in the Comparative Toxicogenomics Database, which included all but 21 of these gene symbols. Of the 1,115 genes present in this database, 330 were associated with TSA, and 785 were not previously reported to be associated with TSA. Interestingly, a search of the GSEA database for overlapping gene sets with the 1,115 pan-cell type TSA-responsive genes revealed no consistent GO terms, but strong overlaps with genes containing highly-conserved motifs near their promoters for transcription factors [23], both known and proposed (Fig 13). There were 257 genes in common between the gene set with this promoter motif and the novel TSA-responsive gene set defined by TempO-Seq. These 257 genes were used to query GO Biological Processes, and strong associations were found with transcription and its regulation, consistent with a treatment that perturbs gene expression. Thus, using TempO-Seq to study a well characterized, broad spectrum HDAC inhibitor has allowed definition of potential mechanisms that could underlie changes in expression that are independent of baseline gene expression differences between cell types.

## Discussion

The TempO-Seq whole transcriptome assay was shown to have the sensitivity to measure as few as ~21 molecules or profile as few as 4 cells or a single cell’s worth of RNA input. It has a specificity of 99.6% for single base differences between RNA targets, which permitted DOs to be designed to measure the whole transcriptome without interference from off-target sequences, and making each measurement independent of the others. Consequently, investigators can optimize panels by adding or removing gene assays, or can formulate small panels of genes as a subset of the whole transcriptome assay and expect to maintain fold difference measurements. Further, changes in off-target transcripts do not impact the measurement of targeted RNAs, increasing the sensitivity of the assay to measure small fold-changes reliably. TempO-Seq also provides sensitive measurement of differential expression of as little as a 1.2-fold difference without false positive measurements of differential expression, and has an excellent correlation with fold change measurements by RNA-Seq.

The fact that TempO-Seq targets a specific RNA sequence suggests that read depth per sample can be lower than in shotgun RNA-Seq because only the targeted genes contribute to a library. For example, there is no need to sequence to higher depth to accommodate the sequencing capacity occupied by ribosomal RNAs or high abundance transcripts such as globin in blood samples. Further, the ability to attenuate high abundance transcripts allows optimization of sequencing space.

Using this technology for MCF-7 cells treated with TSA, we extracted a subset of novel TSA-responsive genes that were also consistent across 3 different cell types from the cMAP database, compiled from Affymetrix microarray data. Extending this analysis to find TSA responses in 5 cell types using TempO-Seq enabled the identification of a 1,136-gene TSA response signature that was in common among these cells, of which 785 genes have not previously been reported in a curated database of toxicogenomics data. This result demonstrates that the whole transcriptome TempO-Seq assay has the precision, accuracy and sensitivity necessary to discover novel treatment-responsive genes. The detection of overlapping gene sets that point to a common mechanism suggests that this assay can provide better understanding of compound effects and mechanisms of action, cellular mechanisms, or disease mechanisms, and to provide robust signatures and measurement of changes in gene expression useful for a variety of applications, including classification of compounds, differentiation of analogs, and identification and evaluation of efficacy or toxicity. Because TempO-Seq is a homogeneous assay (in solution, progressive dilution, add-only assay) that can be fully automated, and because it is a targeted and tunable assay that requires less sequencing capacity, sensitive whole transcriptome profiling of even large cohorts or compound screening samples is feasible. By extension, this also means that complex phenotypes and diseases that require large sample numbers for statistical power can utilize TempO-Seq to define cellular and molecular mechanisms more effectively and efficiently than previously possible.

## Supporting information

S1 Fig Reproducibility of sequencing runs. The log2 read counts for an individual sample are compared between the first and second sequencing run of the same library on the same instrument. Samples were replicates of 100 ng of URR run in parallel on one assay plate (samples 1, 3, and 5) or a second assay plate (samples 2 and 4) using the whole transcriptome DO pool at 21,111-plex. Read depth averaged 1.8M reads per sample in the 1^st^ run and 3.3M reads per sample in the 2^nd^ run.
(TIF)

S2 Fig Behavior of attenuated genes in detecting fold changes. Fold differences between MCF-7 cells and MDA-MB-231 cells measured by TempO-Seq and RNA-seq are compared. Genes that were attenuated to reduce read counts are highlighted in orange.
(TIF)

S3 Fig Back-calculation of attenuated genes. (A) Samples were assayed with or without attenuation and compared. The attenuated genes are highlighted in orange, showing that their read counts are capped. (B) After normalization, the attenuated targets are back-calculated by multiplying by fold attenuation for each target. The back-calculated read counts (orange) are in alignment with the unattenuated population (blue).
(TIF)

S4 Fig Performance of DOs for 46 novel TSA-responsive genes. The cross-platform data from Fig 7 are highlighted to show the 46 TSA-responsive genes detected by TempO-Seq (orange).
(TIF)

S1 Table Reads per sample by input amount. The average number of mapped reads is listed for each level of purified RNA input and each level of cells input as lysates.
(XLSX)

S2 Table TSA cMAP overlaps. The top 10 overlapping profiles in cMAP are shown for a query of the 43 genes that are differentially expressed by TSA across cell types and confirmed in TempO-Seq.
(XLSX)

S3 Table Differentially expressed genes in common for 3 cell types. The table shows the output of DESeq2 for TSA responses from MCF-7, PC-3, and HL-60 cells.
(XLSX)

S4 Table TSA-responsive genes in common across 5 cell types. Genes are listed that have a padj <0.05 for differential expression due to TSA treatment in each of the cell types MCF-7, PC-3, HL-60, HL-60 differentiated with DMSO and HL-60 differentiated with All Trans Retinoic Acid.
(XLSX)
