# Supplementary figures and images for: A trichostatin A expression signature identified by TempO-Seq targeted whole transcriptome profiling
Source: PLoS One. 2017 May 25;12(5):e0178302. doi: 10.1371/journal.pone.0178302 (PMC5444820; doi:10.1371/journal.pone.0178302)

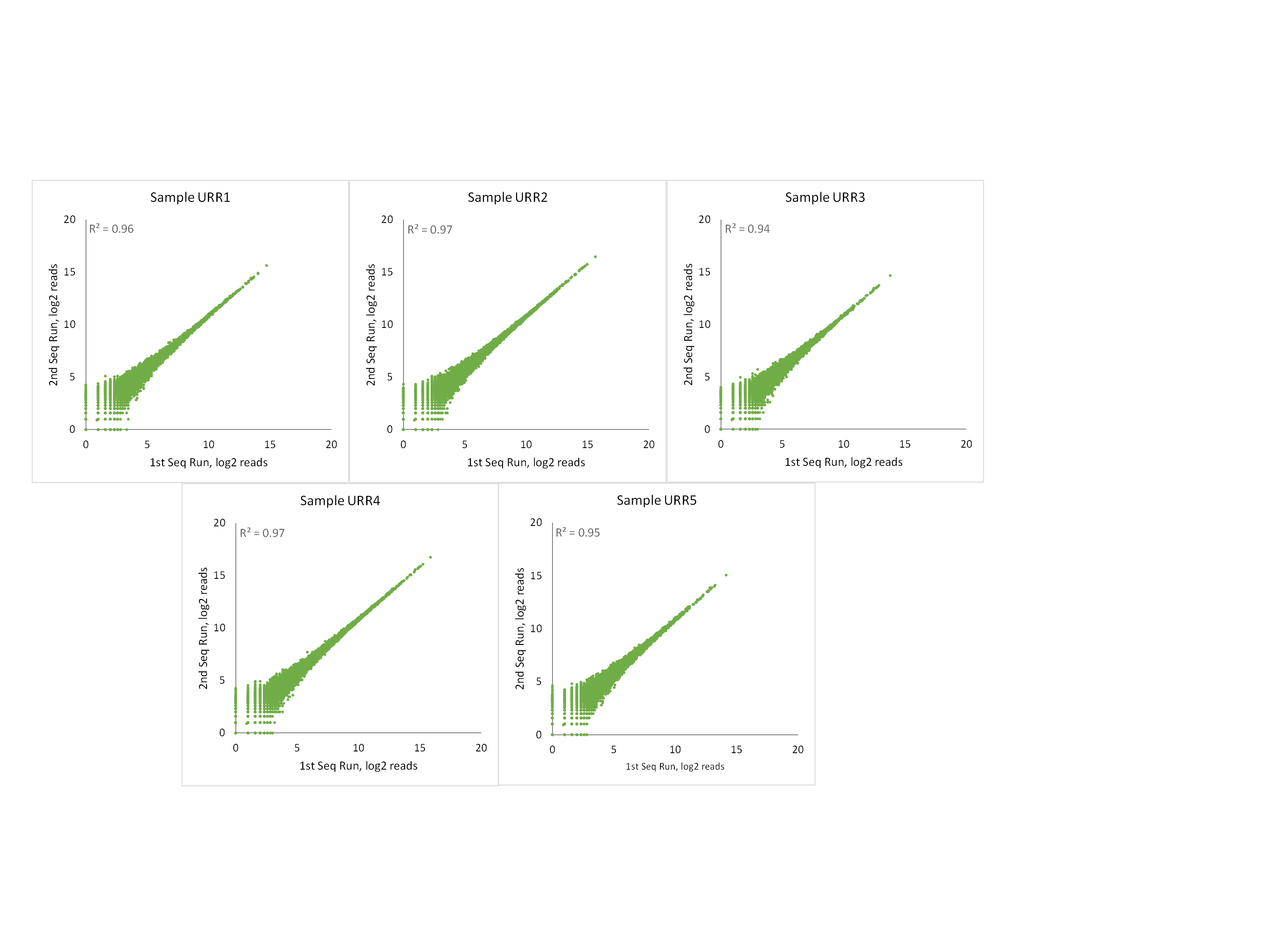

Supplement: S1 Fig — The log2 read counts for an individual sample are compared between the first and second sequencing run of the same library on the same instrument. Samples were replicates of 100 ng of URR run in parallel on one assay plate (samples 1, 3, and 5) or a second assay plate (samples 2 and 4) using the whole transcriptome DO pool at 21,111-plex. Read depth averaged 1.8M reads per sample in the 1st run and 3.3M reads per sample in the 2nd run. (TIF) [file pone.0178302.s001.tif]

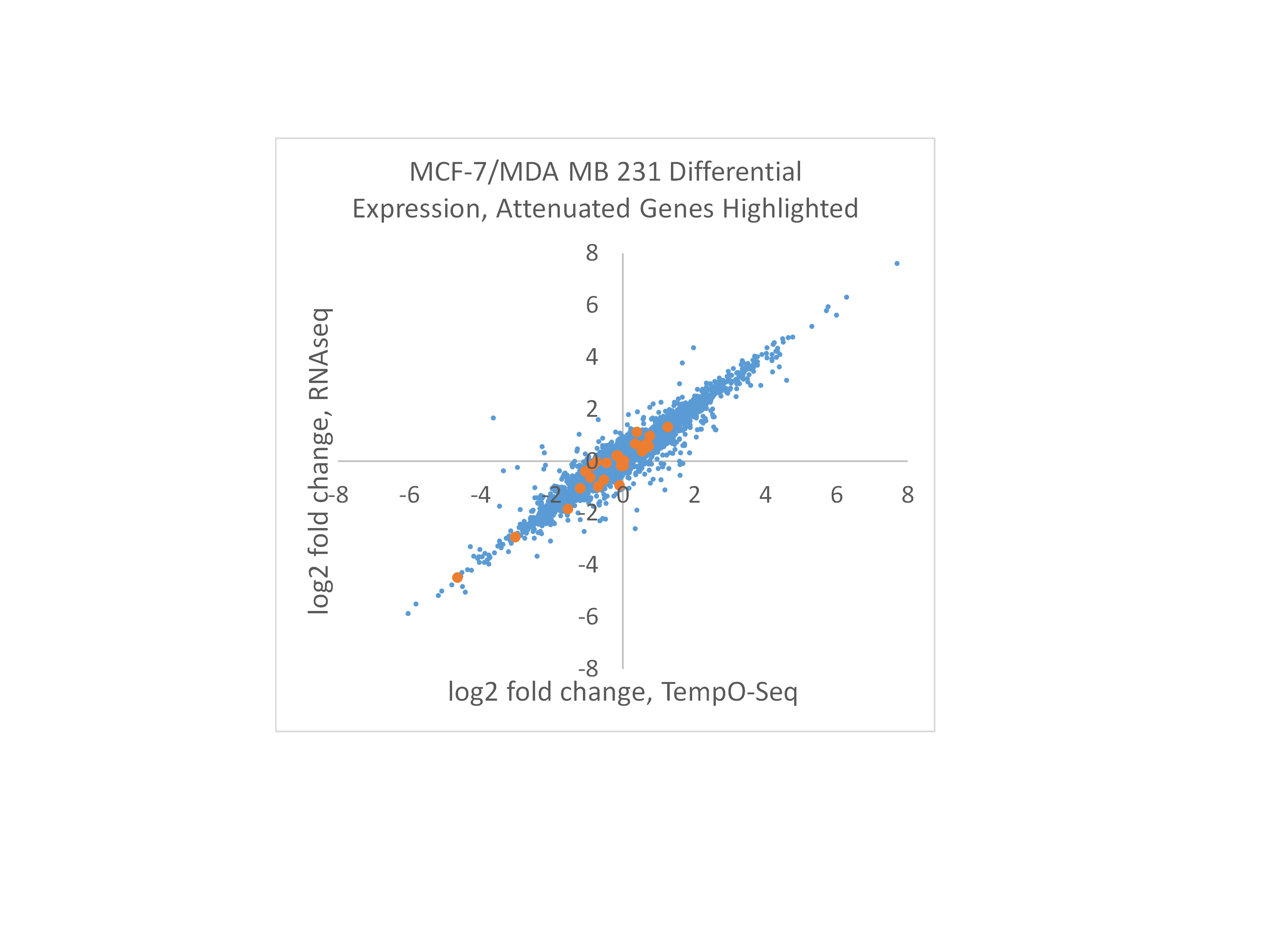

Supplement: S2 Fig — Fold differences between MCF-7 cells and MDA-MB-231 cells measured by TempO-Seq and RNA-seq are compared. Genes that were attenuated to reduce read counts are highlighted in orange. (TIF) [file pone.0178302.s002.tif]

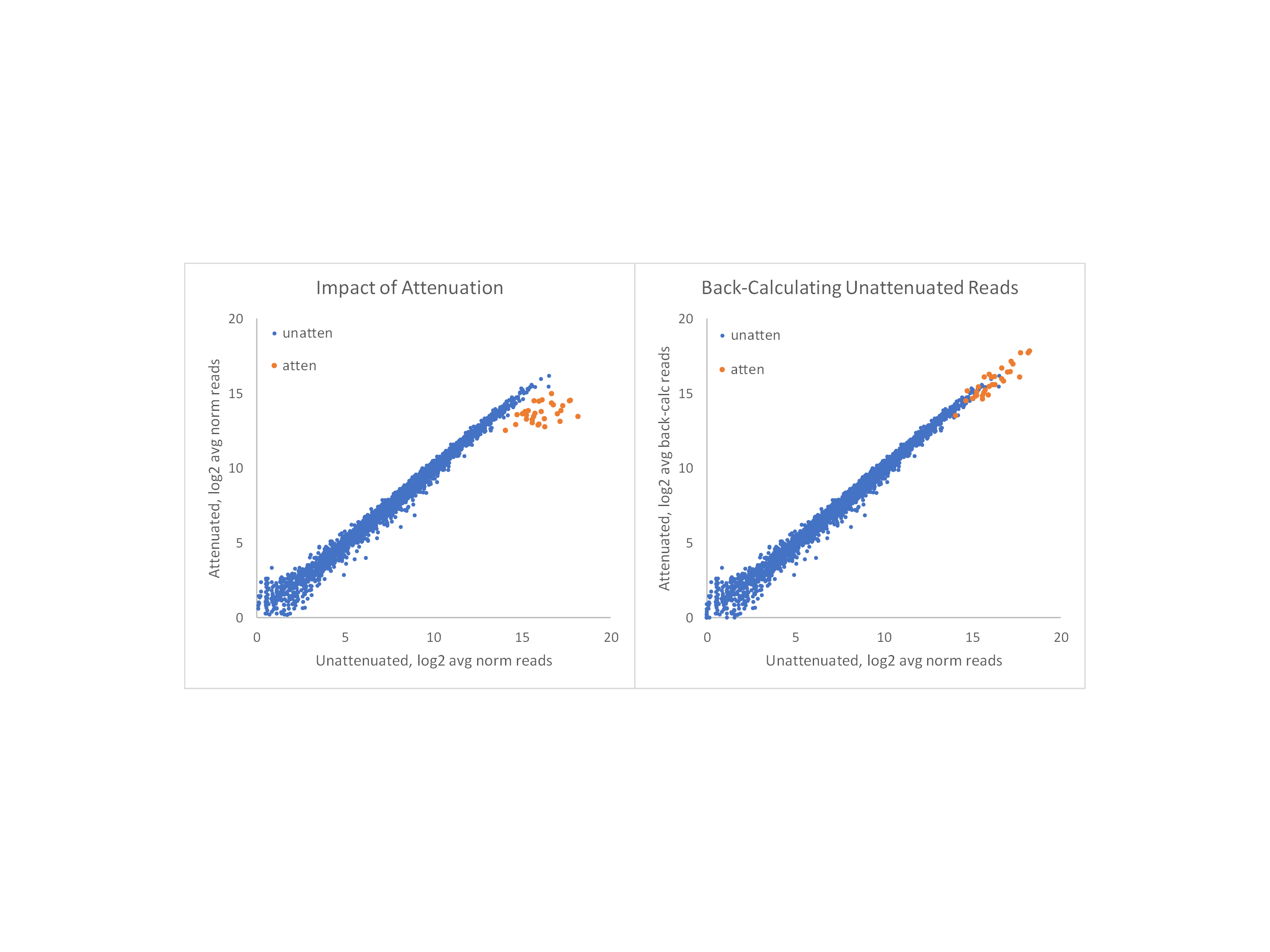

Supplement: S3 Fig — (A) Samples were assayed with or without attenuation and compared. The attenuated genes are highlighted in orange, showing that their read counts are capped. (B) After normalization, the attenuated targets are back-calculated by multiplying by fold attenuation for each target. The back-calculated read counts (orange) are in alignment with the unattenuated population (blue). (TIF) [file pone.0178302.s003.tif]

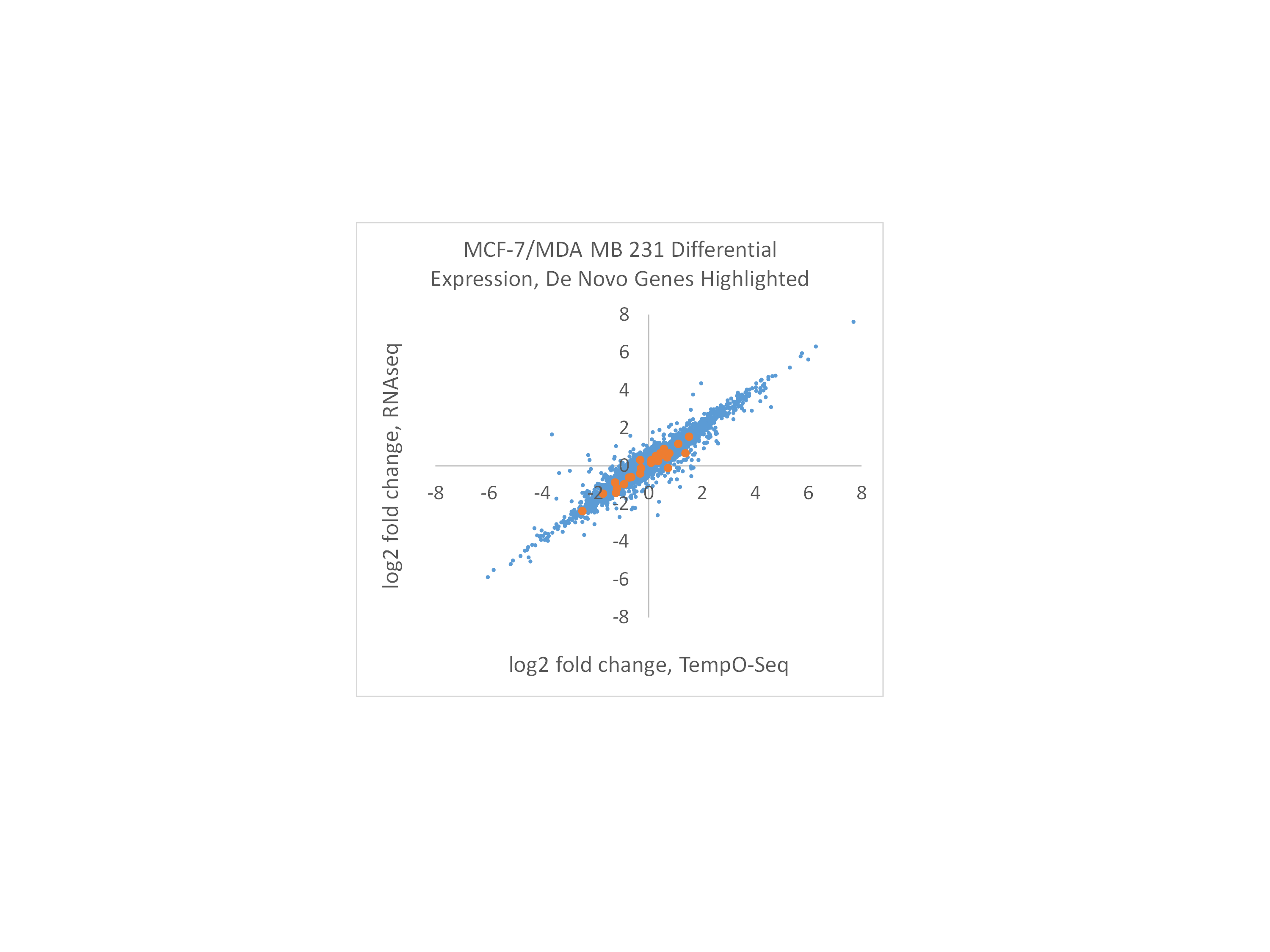

Supplement: S4 Fig — The cross-platform data from Fig 7 are highlighted to show the 46 TSA-responsive genes detected by TempO-Seq (orange). (TIF) [file pone.0178302.s004.tif]
